# Nomogram to determine predictive risk for active tuberculosis based on the QuantiFERON-TB Gold In-Tube test

**DOI:** 10.1038/s41598-023-38900-5

**Published:** 2023-07-24

**Authors:** Qiang Wang, Fengdan Zhu, Yanjuan Cai, Tao Zhu, Xiaolan Lu

**Affiliations:** 1 Department of Clinical Laboratory, Affiliated Hospital of North Sichuan Medical College, Nanchong, Sichuan People’s Republic of China; 2 Department of Laboratory Medicine, North Sichuan Medical College, Nanchong, Sichuan People’s Republic of China; 3 Translational Medicine Research Center, North Sichuan Medical College, Nanchong, Sichuan People’s Republic of China; 4 Department of Laboratory Medicine, Nanchong Central Hospital, Nanchong, Sichuan People’s Republic of China; 5 Department of Preventive Medicine, North Sichuan Medical College, Nanchong, 637000 Sichuan Province China

**Keywords:** Diseases, Infectious diseases, Tuberculosis

## Abstract

Interferon-γ release assay (IGRA) is a widely used blood test for detecting TB infection. However, a positive result of IGRA cannot differentiate active tuberculosis (ATB) infection from inactive tuberculosis (IATB). In this study, we established a nomogram model for predictive risk of ATB, differentiated from IATB, based on the concentration of interferon-γ (IFN-γ) of QuantiFERON-TB Gold In-Tube Test (QFT-GIT) and clinical characteristics. Participants with a positive QFT-GIT result were recruited and divided into a training and validation cohort according to hospitalisation date. The nomogram model for the differential diagnosis of ATB from IATB was established according to gender, age, pleural effusion (PE), and the concentration of IFN-γ in the Nil, TB antigen, and mitogen tube of QFT-GIT in the training cohort by logistic regression and validated in the validation cohort, and then combined with adenosine deaminase (ADA) to evaluated the performance value in ATB cases with PE. The area under receiver operating characteristic curve (AUC) of the diagnostic nomogram model, which we called the NSMC-ATB model for ATB diagnosis was 0.819 (95% CI 0.797–0.841), with sensitivity 73.16% and specificity 75.95% in training cohort, and AUC was 0.785 (95% CI 0.744–0.827), with sensitivity 67.44% and specificity 75.14% in validation cohort. A combination of the NSMC-ATB model and ADA performed better than the NSMC-ATB model and ADA alone in predicting ATB cases with PE, as AUC was 0.903 (95% CI 0.856–0.950) with sensitivity 78.63% and specificity 87.50%. We established an effective diagnostic nomogram model, called the NSMC-ATB model to differentiate ATB from IATB. Meanwhile, the combination of the NSMC-ATB model and ADA improved the performance value of ATB with PE.

## Introduction

Tuberculosis (TB) is an infectious disease that is a major cause of ill health, one of the top 10 causes of death worldwide, and the main cause of death caused by a single infectious agent. In 2020, about 5.8 million new TB cases were reported globally, of which more than 80% were pulmonary TB^1^. Prompt diagnosis and treatment of active tuberculosis (ATB) are essential to ensure better patient outcomes and prevent the transmission in health-care centres and communities^2^. However, there are some difficulties in ATB diagnosis, such as difficulties in obtain a positive culture due in case of low bacterial load and slow culture growth, chest X-ray findings absent or misinterpreted, biopsy material not specific, decreased tuberculin sensitivity, and symptoms and signs of tuberculosis being easily attributed to a pre-existing disease^3^. Thus the diagnosis of suspected TB is lengthy, costly, and burdensome for both patients and health-care systems and results in substantial delays in diagnosis and treatment of other diseases in cases when suspected TB is eventually ruled out^4^. Recently, the progress of molecular diagnosis and other pathogenic microorganism diagnosis technology has improved the speed and accuracy of microbial diagnosis, with high specificity^5,6^. However, the sensitivity of these diagnostic techniques in diagnosing ATB is far from enough, resulting in a substantial delay in diagnosis for a large number of ATB cases^5,6^. Because of the large number of microorganism culture negative TB cases, highly sensitive blood tests are based mainly on the measurement of the immune response to *M. tuberculosis*, rather than the direct detection of bacteria or nucleic acids. These tests help to triage patients in a timely manner at the time of clinical manifestations, addressing a major unmet clinical need that has been prioritised by the World Health Organization^1,7^.

Interferon-γ release assay (IGRA) is a widely used blood test for detecting TB infection^8–10^. IGRA are in vitro assays that rely on CD4 + T cells with cyclic effector memory or central memory phenotypes stimulated by specific TB antigens (including CFP-10, ESAT-6, and TB7. 7), and rapidly produce interferon-γ (IFN-γ) to indirectly detect possible TB infection^11^. Since the *M. tuberculosis* RD1 and 11 regions encoding these specific TB antigens were deleted from the genome of *M. bovis* BCG and are also not present in environmental mycobacteria, IGRA results are less likely to be confounded by BCG vaccination than the tuberculin skin test (TST). Thus, IGRA has an increased diagnostic specificity, a high negative predictive value, and a negative result largely excludes the presence of TB infection^12^.

Currently, two commercial IGRAs, namely T-SPOT.TB (Oxford Immunotec, Abingdon, UK) and QuantiFERON-TB Gold In-Tube Test (QFT-GIT) (Qiagen, Hilden, Germany) are widely used worldwide^13,14^. Since the QFT-GIT’s mitogen stimulation tube is used as a positive control, this test can evaluate the immune response ability and activation function state of the subject's CD4 + T cells after stimulation, it has more advantages in interpreting results^15^. The sensitivity of QFT-GIT for diagnosing TB infection is over 85%. However, a positive result cannot differentiate ATB infection from inactive tuberculosis (IATB—including latent TB infection, LATB; and previous TB infection, PTB) infection, and has a low correlation with the presence of viable bacteria and the risk of developing active disease, which is an important factor in the difficulty of diagnosing ATB^16,17^.

Mpande et al.^18^ suggested that recent *M. tuberculosis* infection is associated with a highly activated and moderately differentiated functional *M. tuberculosis*-specific T cell subset, which can be used as a biomarker to distinguish recent infection from distant infection. In this study, we hypothesised that CD4 + T cells with an effector memory or central memory phenotype in ATB patients and IATB patients have a different effect on the rate and ability to produce IFN-γ upon stimulation with specific TB antigens. According to the widespread application of nomogram in predicting the presence risk of disease^19,20^, in this study, based on the concentration of IFN-γ in the Nil, TB antigen, and mitogen tube of QFT-GIT, combined with other clinical characteristics of patients, we attempted to create a nomogram model for predicting the presence risk of ATB, and to solve the problem of ATB being difficult to distinguish from IATB.

## Materials and methods

### Study setting and patients

Cases with positive QFT-GIT results between January 2019 and March 2021 at the Affiliated Hospital of North Sichuan Medical College were identified, and the 1913 cases were divided as follows: cases from January 2019 to October 2020 were classified as the training cohort, including 637 ATB cases and 790 IATB cases. Cases from November 2020 to March 2021 were classified as the validation cohort, including 172 ATB cases and 314 IATB cases. A diagnosis of ATB needed to meet at least one of the following diagnostic criteria: (1) nucleic acid result for *M. tuberculosis* in sputum or pleural effusion (PE) was positive; (2) sputum smear or culture for *M. tuberculosis* was positive; (3) after treatment for TB, clinical symptoms were significantly relieved or disappeared; (4) the imaging evidence was either significantly absorbed by the lung lesions or the PE volume was significantly reduced after treatment for TB^3^. LATB cases and PTB cases were uniformly included in IATB cases as a control group. LATB was defined as the lack of clinical symptoms, microbial and imaging evidence of ATB, but with a positive QFT-GIT result^21^; PTB was defined as having prior ATB, no recent clinical symptoms, microbial and imaging evidence of ATB after anti-TB treatment^22^.

### QFT-GIT assay

The QFT-GIT assay was performed according to the manufacturer’s instructions (Qiagen, Hilden, Germany). First, 1 ml of whole blood was collected into each of the QFT-GIT blood collection tubes—which included a Nil tube as a blank control tube, TB antigen tube, and a mitogen tube as a positive control. The tubes were shaken to mix the antigen with the blood and incubated at 37℃ ± 1℃. Following a 16-to-24-h incubation period, the tubes were centrifuged, and the final concentration of IFN-γ in plasma was tested by enzyme-linked immunosorbent assay (ELISA). The IFN-γ standard curve was generated for each ELISA plate, and the concentration determined accordingly. The QFT-GIT assay was considered positive when both of the following criteria were met: (1) the IFN-γ of Nil tube ≤ 8.0 IU/mL; (2) the IFN-γ of TB antigen tube minus the IFN-γ of Nil tube ≥ 0.35 IU/mL and ≥ 25% the IFN-γ of Nil tube^23^.

### Other assays

TB DNA was detected by real-time polymerase chain reaction (DAAN GENE Co., Guangzhou, China). Lymphocyte count was detected by BC-6800 (Mindray, Shenzhen, China). The adenosine deaminase (ADA) concentration in PE was measured by peroxidase method (Maccura, Chengdu, China).

### Statistical analysis

After data cleaning, data were imported into the SPSS 19.0 software package, version 19.0 (SPSS Inc., United States) for statistical analysis. Measurement data were described by median (interquartile range), and count data were described by frequency (percentage). The Pearson chi-square test was used to compare count data between groups, and the Mann–Whitney U test was used to compare measurement data between groups. Based on the data of the training cohort, a multivariate predictive risk model for ATB was established using binary logistic regression, and the independent variables were screened via the stepwise method with the inclusion criteria of P < 0.10, and the partial regression coefficient values, odds ratios (OR), 95% confidence intervals (95% CI) of the OR values, and P values of the included independent variables were calculated. The receiver operating characteristic (ROC) curve was used to determine the diagnostic cut-off value, AUC, sensitivity, and specificity. AUCs were compared using Medcalc, version 12.3 (MedCalc Software Ltd, Ostend, Belgium). A nomogram was built using the multivariate analysis results with the rms package of RStudio software. Statistical significance was set as *P* < 0.05.

### Ethical approval and informed consent

This study was approved by the Ethics Committee of Affiliated Hospital of North Sichuan Medical College and conducted in accordance with the declaration of Helsinki Principles. As a retrospective study, informed consent of this study was exempted by the Ethics Committee of Affiliated Hospital of North Sichuan Medical College. The data of this study was collected from the Affiliated Hospital of North Sichuan Medical college and only used for analysis and statistics in this study, and the clinical informations of patients were kept confidential.

## Results

### Characteristics of study population

A total of 1913 patients with positive QFT-GIT results were included in this study and divided into two cohorts, with 1427 cases in the training cohort for the establishment of the diagnostic nomogram model, and 486 cases used as the validation cohort for validating the model. The age of ATB patients in the training and validation cohorts were significantly lower than the IATB patients (*P* < 0.001). ATB and IATB patients were mainly male, with no statistical difference in sex between the two cohorts (*P* > 0.05). Additionally, the levels of IFN-γ in the Nil and TB antigen tubes of ATB patients in the training and validation cohorts were significantly higher than those of IATB patients (*P* < 0.001), while the levels of IFN-γ in the mitogen tube of ATB patients in the two cohorts were significantly lower than those of IATB patients (*P* < 0.001). The proportion of ATB patients with PE in the training and validation cohorts were 33.44% and 27.91%, respectively, which were higher than 20.25% and 19.75% of IATB patients (*P* < 0.001 and* P* = 0.040), respectively. The clinical characteristics of the study population are shown in Table 1.

**Table 1:** Characteristics of the study population (n = 1913).

Characteristic	Training cohort		Validation cohort	
	Active PTB (n = 637)	Inactive PTB (n = 790)	Active PTB (n = 172)	Inactive PTB (n = 314)
Age (years)	48 (26–63)*	56 (47–68)	46 (25–57)^#^	57 (48–68)
Gender				
Male, n (%)	401 (62.95%)	471 (59.62%)	107 (62.21%)	199 (63.38%)
Female, n (%)	236 (37.05%)	319 (40.38%)	65 (37.79%)	115 (36.62%)
IFN-γ (IU/mL)				
Nil	0.160 (0.060–0.430)*	0.070 (0.030–0.200)	0.315 (0.130–0.785)^#^	0.140 (0.050–0.425)
TB antigen	6.400 (2.885–11.295)*	2.570 (1.118–6.343)	5.095 (2.060–9.940)^#^	1.950 (0.945–5.985)
Mitogen	7.180 (2.160–13.670)*	10.185 (5.008–16.093)	9.090 (3.545–21.250)^#^	13.055 (5.220–25.855)
Lymphocyte (per nl)	1.26 (0.87–1.71)	1.40 (1.01–1.81)	1.20 (0.84–1.70)	1.47 (1.11–1.87)
Pleural effusion				
Yes, n (%)	213 (33.44%)*	160 (20.25%)	48 (27.91%)^#^	62 (19.75%)
No, n (%)	424 (66.56%)	630 (79.75%)	124 (72.09%)	252 (80.25%)
TB DNA				
Positive, n (%)	256 (40.19%)	NA	99 (57.56%)	NA
Negtive, n (%)	381 (59.81%)	790 (100.00%)	73 (42.44%)	314 (100.00%)

Data are expressed as median (interquartile range) or number (%).

*NA* Not applicable.

*P < 0.05, compared with IATB in training cohort.

^#^*P* < 0.05, compared with IATB in validation cohort.

### Diagnostic nomogram model for ATB

Data correction and conversion: based on training cohort data, the levels of IFN-γ in the Nil, TB antigen, and mitogen tube were calibrated by the ratio to lymphocyte count, and then the natural logarithm of the calibrated levels of IFN-γ and age were performed as ln(Nil/Lymphocyte), ln(TB antigen/Lymphocyte), ln(mitogen/Lymphocyte), and ln(years).

**Figure 1:**
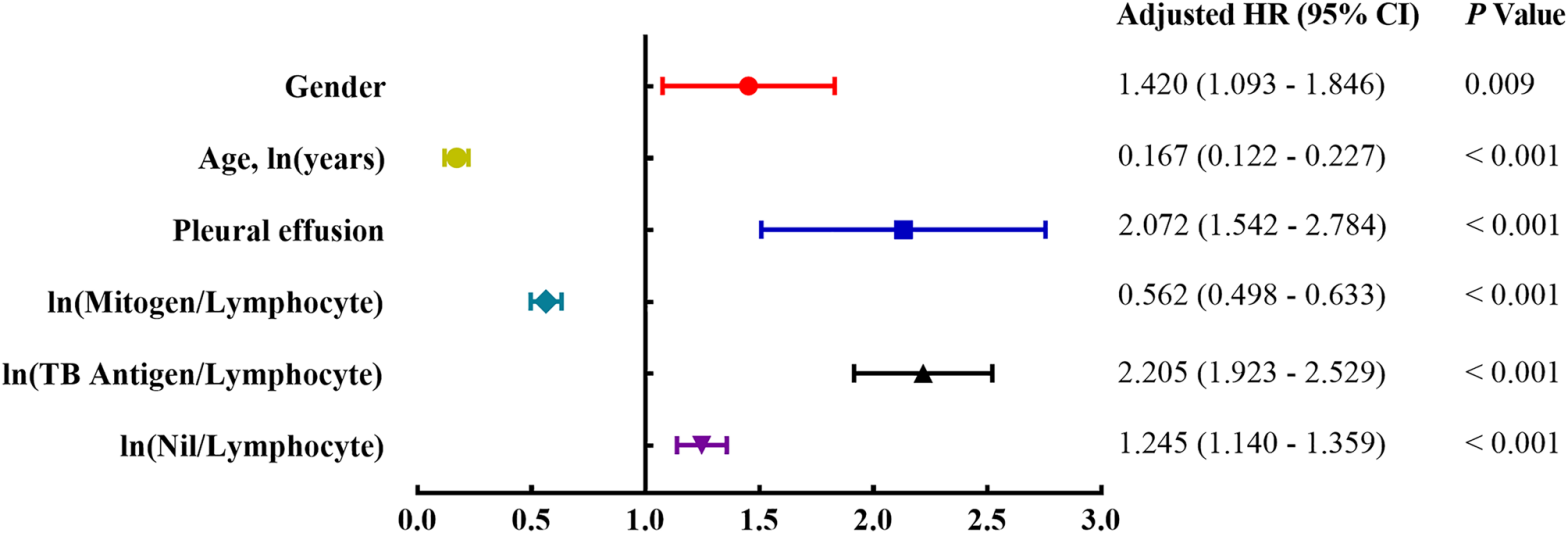
Forest plot of variables in the diagnosis of ATB.

Taking ATB as the dependent variable and ln(Nil/Lymphocyte), ln(TB antigen/Lymphocyte), ln(mitogen/Lymphocyte), ln(years), gender, and PE as independent variables for binary logistic regression analysis. The code for each variable were: gender (male = 1, female = 0) and PE (yes = 1, no = 0). The results of multivariate logistic regression analysis showed that ln(Nil/Lymphocyte), ln(TB antigen/Lymphocyte), PE, and male were risk factors for ATB, while ln(mitogen/Lymphocyte) and age were protective factors for ATB (Fig. 1).

**Figure 2:**
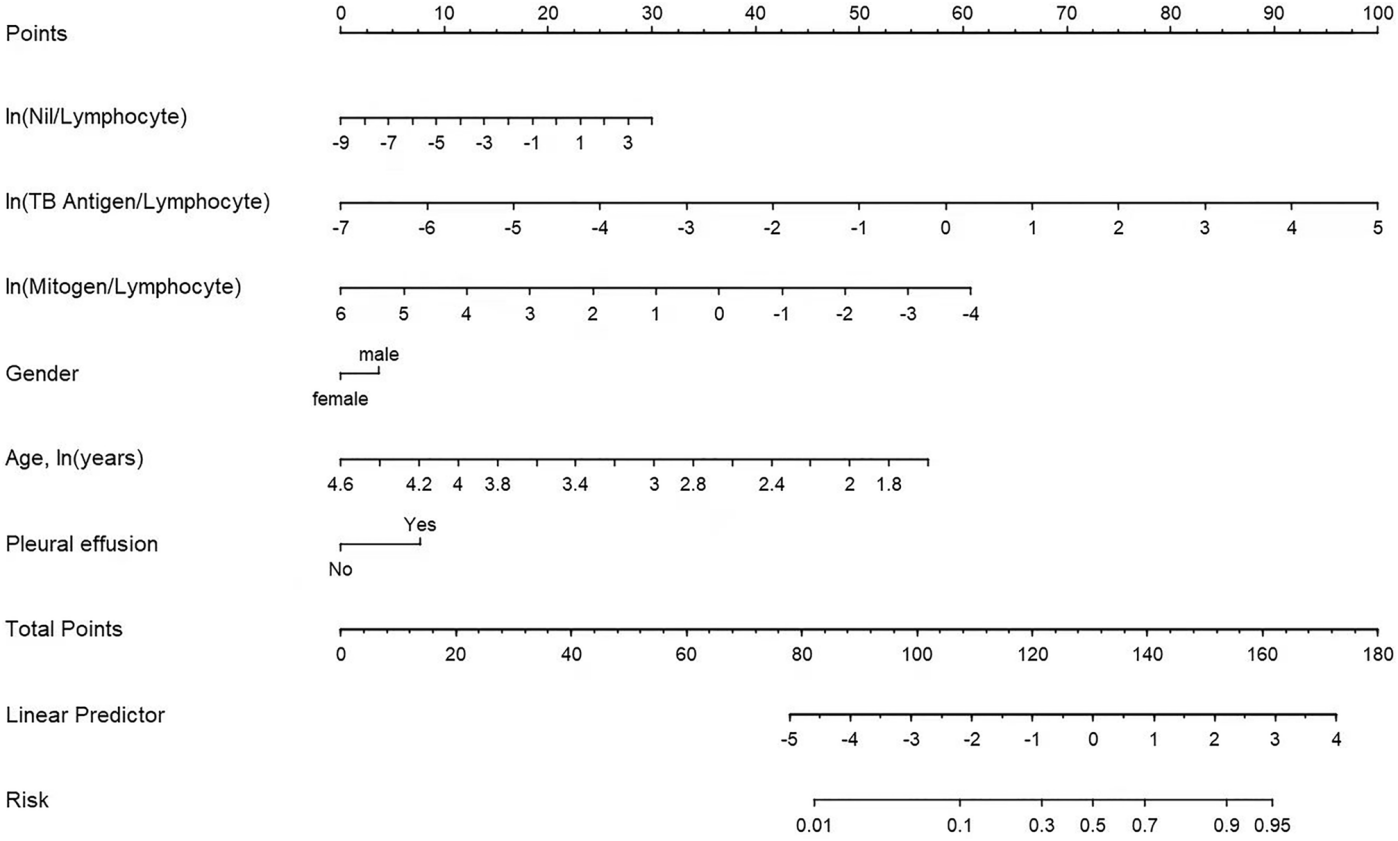
Nomogram to predict the presence of ATB. To use the nomogram, draw an upward vertical line to the “Points” bar to obtain the points of each variables. Based on “Total Points” summed by points of each variables, draw a downward vertical line from “Total Points” to the “Risk” bar to obtain the risk of the presence of ATB.

According to the multi-factor predictive risk variables in the training cohort a diagnostic nomogram model—which we called the NSMC-ATB model—was created to predict the risk of developing ATB (Fig. 2). The calculated risk of the NSMC-ATB model was based on the model formula: ln(p/1-p) = 6.814 + 0.351 × gender–1.791 × ln(age) + 0.728 × PE + 0.219 × ln(Nil/Lymphocyte) + 0.791 × ln(TB antigen/Lymphocyte)–0.577 × ln(mitogen/Lymphocyte).

### Diagnostic performance of NSMC-ATB model in ATB

**Figure 3:**
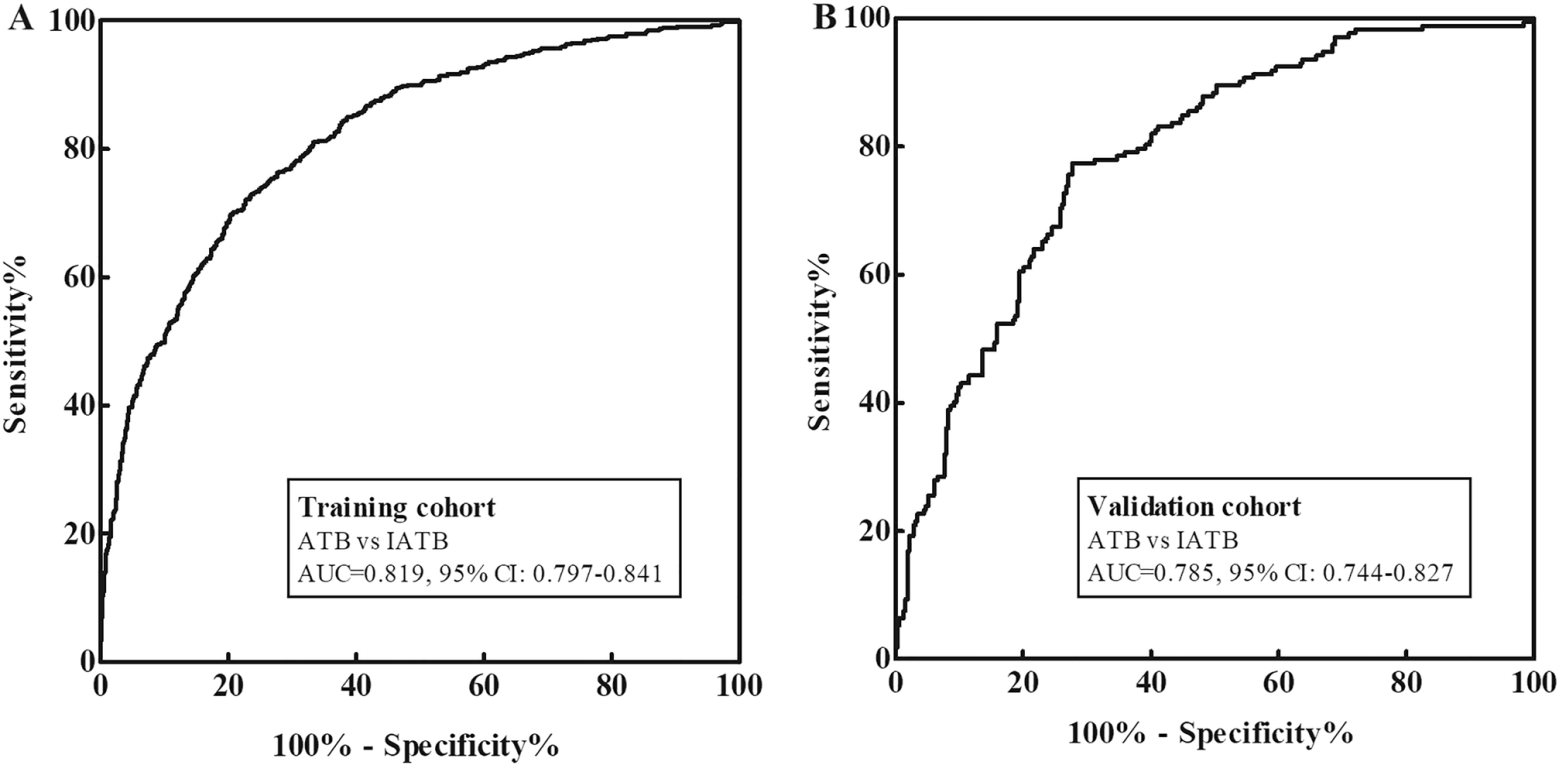
AUCs of the NSMC-ATB model to discriminate patients with ATB from IATB. (**A**) Training cohort; (**B**) Validation cohort.

In the training cohort, 790 IATB cases as control and the cut-off value was set at 0.449, the AUC of the NSMC-ATB model for 637 ATB cases diagnosis was 0.819 (95% CI 0.797–0.841; Fig. 3A), sensitivity 73.16%, specificity 75.95%, and accuracy 74.70%. The AUCs of the NSMC-ATB model for 256 ATB cases with TB DNA-positive diagnosis and 381 ATB cases with TB DNA-negative diagnosis were 0.816 (95% CI 0.785–0.846) and 0.821 (95% CI 0.796–0.847), respectively, but the difference between them was not statistically significant (*P* = 0.808). When combined with the TB DNA assay, the sensitivity of the NSMC-ATB model for ATB diagnosis could be improved from 73.16% to 83.99% (Table 2).

**Table 2:** The novel diagnostic model, TB DNA, or both in the diagnosis of active PTB.

	Cut-off value	Training cohort					Validation cohort				
		AUC (95% CI)	SEN (%)	SPE (%)	PPV (%)	NPV (%)	AUC (95% CI)	SEN (%)	SPE (%)	PPV (%)	NPV (%)
NSMC-ATB model											
ATB vs. IATB	0.449	0.819 (0.797–0.841)	73.16	75.95	71.04	77.82	0.785 (0.744–0.827)	67.44	75.14	59.79	80.82
ATB with TB DNA-positive vs. IATB with TB DNA-positive		0.816 (0.785–0.846)	73.05	75.95	49.60	89.69	0.798 (0.750–0.846)	71.72	75.14	47.65	89.39
ATB with TB DNA negtive vs. IATB with TB DNA negtive		0.821 (0.796–0.847)	73.23	75.95	59.49	85.47	0.768 (0.710–0.826)	61.64	75.14	36.59	89.39
NSMC-ATB + TB DNA											
ATB vs. IATB		0.893 (0.876–0.910)	83.99	75.95	73.79	85.47	0.902 (0.872–0.932)	83.72	75.14	64.86	89.39

*SEN* Sensitivity; *SPE* Specificity; *PPV* Positive predictive value; *NPV* Negative predictive value.

We validated the NSMC-ATB model using the validation cohort. Using 314 IATB cases as control, the AUC of the NSMC-ATB model for 172 ATB cases diagnoses was 0.785 (95% CI 0.744–0.827; Fig. 3B), which compared to the training cohort AUC, the difference was not statistically significant (*P* = 0.152). The AUCs of the NSMC-ATB model for 99 ATB cases with TB DNA-positive diagnosis and 73 ATB cases with TB DNA-negative diagnosis were 0.798 (95% CI 0.750–0.846) and 0.768 (95% CI 0.710–0.826), respectively, and the difference was not statistically significant (*P* = 0.442). When combined with the TB DNA assay, the sensitivity of the NSMC-ATB model for ATB diagnosis was improved from 67.44% to 83.72% (Table 2).

### Diagnostic performance of the NSMC-ATB model combined ADA in ATB with PE

**Figure 4:**
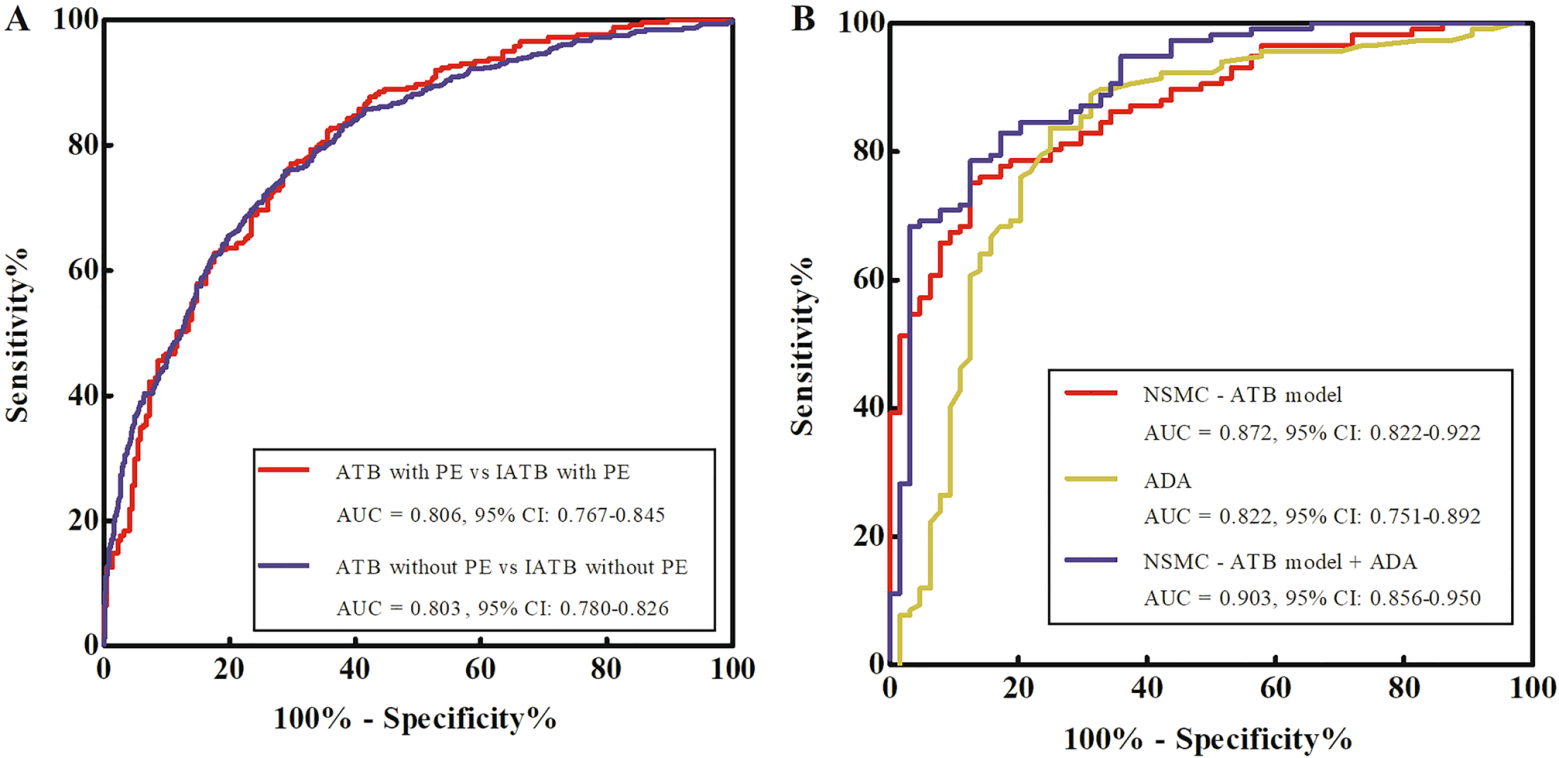
(**A**) Comparation of AUCs of the NSMC-ATB model between in ATB cases with PE diagnosis and in ATB cases without PE diagnosis. (**B**) Comparation of AUCs of the NSMC-ATB model, ADA, or both in ATB cases with PE diagnosis.

There were a total of 483 cases with PE in the training and validation cohorts, including 261 ATB and 222 IATB cases, and a total of 1430 cases without PE, including 548 ATB and 882 IATB cases. The NSMC-ATB model had an AUC of 0.806 (95% CI 0.767–0.845) for ATB cases with PE diagnosis, compared with the AUC of 0.803 (95% CI 0.780–0.826) for ATB cases without PE diagnosis, and the difference was not statistically significant (*P* = 0.864; Fig. 4A).

Among the cases with PE, a total of 181 cases had completed the detection of ADA concentration in PE, including 117 ATB and 64 IATB cases. The AUC of the NSMC-ATB model combined with ADA in the diagnosis of ATB with PE was slightly improved (0.903 vs. 0.872 and 0.822), although there was still no statistical difference (*P* = 0.371 and 0.061; Fig. 4B and Table 3).

**Table 3:** The novel diagnostic model, ADA, or both in the diagnosis of active PTB with PE.

	AUC (95% CI)	SEN (%)	SPE (%)	PPV (%)	NPV (%)
NSMC-ATB model	0.872 (0.822–0.922)	75.21	87.50	91.67	65.88
ADA	0.822 (0.751–0.892)	83.76	75.00	85.96	71.64
NSMC-ATB + ADA	0.903 (0.856–0.950)	78.63	87.50	92.00	69.14

### Proposed risk scale

According to the data of the training and validation cohorts, we proposed a simple standardised scale of predictive risk for clinicians to evaluate the probability of ATB (Table 4), which is mainly based on the following principles: (1) “low risk” was defined as the predictive risk being lower than the maximum predictive risk of negative predictive value (NPV)  ≥  90.00%; (2) “moderate risk” was defined as the predictive risk between the minimum predictive risk of NPV  <  90.00% and  <  cut-off value.; (3) “high risk” was defined as the predictive risk between  ≥  cut-off value and the maximum predictive risk of positive predictive value (PPV)  <  90.00%; (4) “most likely ATB” was defined as the predictive risk being higher than the minimum predictive risk of PPV  ≥  90.00%.

**Table 4:** Proposed risk scale and corresponding predictive risk of ATB.

Risk level	Predictive risk	PPV (%)	NPV (%)
Low risk	0.000–0.161	NA	90.13–100.00
Moderate risk	0.162–0.448	NA	78.58–89.90
High risk	0.449–0.871	68.31–89.73	NA
Most likely ATB	0.872–1.000	90.34–100.00	NA

*NA* Not applicable.

## Discussion

The problem of the accurate and timely diagnosis of ATB has plagued clinicians for a long time, since the sensitivity of etiological detection is only about 50%^1^. Although this problem has been solved within a certain sensitivity range through the continuous development and improvement of diagnostic technology and methods, effective diagnosis remains a challenge^5,24–26^. In this study, we established a diagnostic nomogram model, which we called NSMC-ATB model, to predict the presence of ATB by combining gender, age, PE, and corrected levels of IFN-γ in the Nil, TB antigen, and mitogen tubes of the QFT-GIT. Through evaluation using a training and validation cohort, this model was shown to have a good sensitivity and specificity in ATB diagnosis. Additionally, combined with ADA, the diagnostic performance of the NSMC-ATB model in ATB with PE has been further improved. Therefore, in the absence of etiological evidence, the NSMC-ATB model can help clinicians more accurately diagnose ATB.

The level of IFN-γ in the TB antigen tube of QFT-GIT is not related to the severity of TB, but is related to infection activity, and the level of IFN-γ stimulated by TB antigen is as follows: patients with residual TB lesions  <  patients with low TB activity  <  patients with high TB activity; mitogen-induced IFN-γ secretion was negatively correlated with lung pathological morphology and the area of affected lung tissue^26,27^. In this study, IFN-γ levels in the Nil and TB antigen tubes in ATB patients in the training and validation cohorts were significantly higher than those in IATB, while IFN-γ levels in the mitogen tube were significantly lower than those in IATB, which was consistent with the conclusions reported by Nikitina et al.^27^. Thus it was feasible to diagnose ATB by the differential expression of IFN-γ in ATB and IATB by QFT-GIT. TB most often infects males^1^, and patients with ATB are significantly younger than those with IATB, which should be related to the disease process of ATB and the transformation into LATB or PTB after completing treatment. IFN-γ is produced by CD4 + T cells with effector or central memory phenotypes. Due to the lack of corresponding experimental data on this type of cell, in this study, we used the ratio of IFN-γ to lymphocyte count to correct the levels of IFN-γ, and logistic multivariate regression analysis was performed on the corrected levels of IFN-γ in the Nil, TB antigen and mitogen tubes, as well as other variables such as gender, age, and PE and the risk variables of ATB were screened out. Based on those risk variables, a diagnostic nomogram model, called the NSMC-ATB model for ATB was built. Through evaluating the training and validation cohorts, the NSMC-ATB model has good accuracy in ATB diagnosis. The sensitivity of TB DNA in ATB in the training and validation cohorts was about 50%, which was consistent with the etiological detection rate of ATB reported by the WHO^1^. There was no statistical difference between the AUCs of the NSMC-ATB model for diagnosing ATB cases with TB DNA-positive and ATB cases with TB DNA-negative, indicating that the NSMC-ATB model did not have a significant relationship with the severity of ATB. The NSMC-ATB model combined with TB DNA can effectively address the lack of diagnostic sensitivity of TB DNA alone, and significantly improves the detection rate of ATB.

Adenosine deaminase (ADA) is the most used biomarker for the diagnosis of tuberculous PE^28^. A meta-analysis indicated that the pooled sensitivity and specificity of ADA for the diagnosis of tuberculous PE were 92% and 90%, respectively^29^. Our results were lower than the data obtained from the meta-analysis, but it was consistent with the results of ADA in the diagnosis of tuberculous PE with malignant PE as reported by Li et al.^30^. The control group in this study was the IATB population with a positive QFT-GIT result, and this is probably the main reason for the differences between our results and those of previous studies. The AUCs of the NSMC-ATB model in ATB cases with and without PE were fairly consistent, while the AUC of the NSMC-ATB model in 117 cases of ATB with PE diagnosis was slightly higher than that in total cases of ATB with PE, although this was not statistically significant, this could be caused by the small number of cases with PE with large sampling error. The AUC of the NSMC-ATB model combined with ADA in the diagnosis of ATB with PE was higher than that of the model alone, indicating that ADA could be used as a supplementary detection for the NSMC-ATB model in ATB cases with PE diagnosis.

Additionally, we quantified the possibility of the presence of ATB, and the risk of ATB can be assessed according to predictive risk calculated by the NSMC-ATB model, which is helpful for clinicians to evaluate and interpret the status of TB infection with negative etiological evidence, and implement timely and reasonable treatment: (1) Low risk. LATB or PTB infection and without TB treatment is recommended. (2) Moderate risk. Repeat etiological tests and priority treatment of other diseases is recommended. (3) High risk. Repeat etiological tests and priority TB treatment selection is recommended. (4) Most likely ATB. Priority TB treatment selection is recommended, even if no etiological evidence is observed.

In conclusion, the NSMC-ATB model can accurately predict the presence of ATB, and successfully differentiate it from LATB and PTB. This model performs well in both the training and validation cohort, which can help clinicians estimate the individual risk of ATB in patients with positive QFT-GIT results, especially those who’s countries are burdened by TB—i.e., India, Indonesia, Philippines, Pakistan, Nigeria, Bangladesh, South Africa, and China—many of which are still developing economically and have rural populations which do not have easy access to medical care.

## Data Availability

The datasets generated during and analyzed during the current study are available from the corresponding author on reasonable request.
